# A twinning initiative between midwife associations in Ghana and Sweden -A process evaluation study

**DOI:** 10.1080/16549716.2025.2457824

**Published:** 2025-02-03

**Authors:** Hanna Fjellström, Emelie Sandberg, Johanna Blomgren, Malin Grahn, Fredrica Hanson, Netta Ackon, Gifty Baidoo, Louise Nordström, Malin Bogren

**Affiliations:** a Institute of Health and Care Sciences, Sahlgrenska Academy, University of Gothenburg, Gothenburg, Sweden; b Department of Women’s and Children’s Health, Karolinska Institutet, Stockholm, Sweden; c Youth Clinic Olskroken, Gothenburg, Sweden; d Ghana Registered Midwives Association, Greater Accra, Ghana; e BB Karolinska Solna C5:35, Eugeniavägen, Solna, Sweden

**Keywords:** Twinning, midwife associations, implementation science, Western Africa, Northern Europe

## Abstract

**Background:**

This study describes the evaluation of a twinning initiative between the Ghana Register Midwives Association and the Swedish Association of Midwives. Recognising the importance of midwives being supported by a national midwife association, the initiative was to strengthen the professional association in Ghana as a labour union and to inspire Swedish midwives to involve themselves in international work.

**Objective:**

The study aimed to evaluate a twinning initiative between the Ghana Registered Midwives Association and the Swedish Association of Midwives.

**Method:**

Two focus group discussions and four individual interviews were held with nine midwives from the Ghana Registered Midwives Association (*n* = 6) and the Swedish Association of Midwives (*n* = 3). The interviews and analysis were guided by a process evaluation framework using content analysis.

**Results:**

The twinning initiative was successfully implemented regarding fidelity, dose, and reach, despite adaptations to the original project plan. Both associations gained visibility, with the Ghana Registered Midwives Association growing its paid membership by 97%, from 631 to 1,245 members during the twinning initiative. The results suggest that the Swedish Association of Midwives enhanced its understanding of international midwifery, promoted knowledge exchange, and raised awareness of midwives’ global roles in improving care.

**Conclusion:**

The Ghana Registered Midwives Association and the Swedish Association of Midwives had a positive experience with the twinning initiative, despite deviations from the original plan. Midwives from both associations benefitted from sharing best practices and mutual support in their roles as newly formed labour trade unions. These findings could benefit other midwife associations in future twinning initiatives.

## Paper context

**Main findings:** Twinning is a useful strategy to increase organisational capacity and membership rates.

**Added knowledge:** Twinning can increase knowledge exchange between association members and make midwives present where decisions occur.

**Global health impact for policy and action:** More twinning between professional associations should be initiated as twinning can foster knowledge exchange, form partnerships, and have the potential to address healthcare quality challenges.

## Background

Evidence recognises that when midwives are educated to international standards, including the provision of family planning, more than 80% of maternal and neonatal deaths can be prevented [1]. Midwives can significantly contribute to improving sexual, reproductive, and maternal health and reducing maternal and newborn mortality, but only if they are adequately educated, regulated, and supported by a professional association [2].

Professional midwife associations are crucial in advocating for enhancements in midwifery education, regulations, and working conditions [3]. Thus, having robust and active midwife associations is essential [4]. The International Confederation of Midwives (ICM) argues that twinning between midwife associations is one strategy to strengthen the midwife profession and associations [5].

Twinning is a collaborative partnership model emphasising reciprocity and mutual learning between two organisations, often from different countries or contexts [6]. Twinning in healthcare further entails building personal relationships which can empower healthcare professionals and strengthen healthcare systems [4,5]. Unlike traditional approaches such as technical assistance (TA), which often involves a unidirectional flow of knowledge and resources from high- to low-income settings, twinning fosters an equitable exchange where both partners share expertise and benefit equally. It is commonly used to build capacity and sustainably improve health outcomes, often between health institutions, professional associations, or academic entities [7,8]. Compared to other concepts like capacity building, North-South partnerships, and peer-to-peer learning, twinning stands out for its focus on long-term, bidirectional collaboration. While capacity building and North-South partnerships often face critiques of power imbalances, twinning aims to create equal partnerships with shared leadership and joint ownership of outcomes. Similarly, although peer-to-peer learning also involves mutual exchange, it typically occurs at an individual or small-group level rather than institutionally [9]. By promoting equity and sustainability, twinning represents a progressive and inclusive approach to global health collaboration [5].

With its intrinsic importance in maternal and child health, the midwife profession is no exception to the potential benefits of twinning initiatives. By uniting midwives from different regions and backgrounds, twinning initiatives have shown promise in augmenting midwives’ capacity, thereby improving maternal and child health outcomes [4].

This paper describes the implementation and evaluation of a twinning initiative between two midwife associations, the Ghana Register Midwives Association (GRMA) and the Swedish Association of Midwives (SBF), and the associations’ twinning. Ghana, one of the partner countries in the twinning initiative, battles with a maternal mortality ratio of 263 per 100,000 live births [10] and a neonatal mortality rate of 23 per 1,000 live births [11]. While there has been a decline in maternal and neonatal mortality rates, significant challenges persist, including limited access to quality intrapartum care, emergency obstetric care, safe abortions, and family planning [12]. Sweden, the second partner country in this initiative, has a maternal mortality ratio of 5 per 100,000 live births [13] and one neonatal death per 1,000 live births [14]. Although Sweden has low maternal and neonatal ratios, midwives report unreasonably high demands, a lack of influence and recognition at work and, in addition, high role conflict and burnout compared to Swedish benchmarks [15].

To gain a deeper understanding of twinning and the processes and working methods applicable to a twinning initiative, this study aimed to evaluate the twinning initiative between the Ghana Registered Midwives Association and the Swedish Association of Midwives

## Method

### Study design

A qualitative research design was used. Data were collected from focus group discussions (FGD) and individual interviews with midwives from Ghana and Sweden who were directly involved in the initiative. This study adhered to the process evaluation guidelines outlined by the Medical Research Council [16], which provides a framework for investigating and assessing four main areas: (1) implementation activities (including dose, fidelity, adaptations, and reach); (2) mechanisms of impact; (3) contextual factors influencing the intervention; and (4) intervention outcomes. The contextual factors influencing the project will be described elsewhere, while this study describes the implementation activities, mechanisms of impact, and outcomes of the twinning initiative.

### Setting

GRMA is a non-governmental association for midwives in Ghana, which was formed in 1935 and is a member of ICM. In 2019, GRMA became a full-fledged labour union with members across all ten regions of Ghana. The association aims to promote sexual and reproductive health and rights, improve healthcare accessibility, expand the scope of midwives’ work, and safeguard midwives’ interests and welfare nationwide. GRMA works to advance the midwifery profession through education and collaborative initiatives, aiming to enhance the quality of care in Ghana in both private and public sectors [17].

SBF was formed in 1886 and became a labour union in 2019, just like GRMA. SBF is a free-standing professional association for midwives to address working conditions and professional development for Swedish midwives. The association influences the development and strengthening of professional competence through education and research in midwifery and reproductive, perinatal, and sexual health and rights. It provides expert knowledge to policymakers and national authorities [18].

### Intervention – the twinning initiative

The three-year twinning initiative between GRMA and SBF started with a workshop in 2020, during which the needs and objectives of the associations were outlined. Both associations wanted to increase their visibility to attract new members and keep the current ones engaged. In addition, the associations had different objectives for the project. SBF aimed to foster greater interest in international midwifery among Swedish midwives and students, gain valuable insights from global midwifery practices and promote a general understanding of the critical need for midwives. GRMA, in turn, identified the need to enhance its administrative capacity and expand membership in order to qualify as a full labour union with the ability to negotiate salaries and working conditions for its members. Given that SBF had recently attained these rights, it was suggested that they share their experiences to support GRMA in achieving these goals. To assist GRMA in meeting its objectives, the associations employed The International Confederation of Midwives (ICM) organisational tool – the Midwifery Association Capacity Tool (MACAT), to develop a strategic plan [19].

The plan included (i) strengthening GRMA’s administrative capacity to function effectively as a professional trade union, (ii) improving membership services through a functioning website and regular newsletters to reach out to members and increase their income as a result of the increased number of paying members; (iii) strengthening the capacity of the executive board members and the junior midwife leaders, as well as (iv) strengthening the members’ ability to advocate for the profession.

The number of group members in the twinning initiative varied between GRMA and SBF, depending on the two associations’ needs, wishes, and feasibility. GRMA chose to include all the council members in managing the twinning initiative. For SBF, the group was kept small due to the limited administrative resources and time allocated to the project. The target group was primarily the council members of GRMA and representatives from ten regions across Ghana, as well as midwives and midwifery students in Ghana and Sweden.

### Participants and data collection

All 15 participants from GRMA (*n* = 11) and the SBF (*n* = 4) project groups were invited to participate in the study. For the participants from Ghana, the General Secretary at GRMA was responsible for disseminating information about participation in either an FGD or an individual interview based on their preference of meeting at the GRMA office in Accra, Ghana, or online. The four Swedish midwives were asked via email to participate in an online FGD, and information about the study was sent out beforehand.

Four (*n* = 4) Ghanaian midwives participated in individual interviews and two (*n* = 2) participated in a FGD. Three of them attended individual in-person interviews at the GRMA office in Ghana, and one attended an individual interview online through Zoom. Of the six midwives who were invited to the FGD online, two (*n* = 2) showed up. The other four later communicated that even though they were able to enter the online meeting, they could not hear or speak during it. The interviews in Ghana were held in English, one of Ghana’s official languages [20]. Three (*n* = 3) out of four SBF midwives joined the FGD, and the fourth communicated that she could not attend due to conflicting activities. The FGD with the Swedish midwives was held in Swedish online through Zoom. The midwives who participated ranged between 33 and 63 years old and had between 3.5 and 39 years of experience working as midwives.

Data was collected in November 2022 using an interview guide, (see Appendix 1), following the structure of process evaluation: implementation process, mechanisms of impact, and outcomes of the implementation [21]. The research questions are presented in detail in Table 1. One interviewer and one observer conducted all interviews and FGDs. The interviews and FGDs took 30–60 minutes and were audio-recorded. Descriptive data on membership numbers in GRMA were collected to show the project’s impact.

**Table 1.** Research questions in relation to the components of the process evaluation in the twinning initiative.

Implementation Process
*Fidelity*
Was the intervention of the project delivered?
*Dose*
To what extent was the project achieved? To what extent did the project result change? Why?
*Adaptations*
Were there any alterations made to achieve a better contextual fit?
*Reach*
To what extent were the midwives involved in the project? Could the midwives’ involvement have been improved?
Mechanisms of Impact of the Implementation
Was the project acceptable and perceived as relevant? What challenges were encountered during the project?
Outcomes of the Implementation
To what extent did the membership increase?

### Data analysis

The principles of deductive content analysis [22] were employed to direct the analysis of the FGDs and interviews. Initially, all transcripts underwent multiple readings. Next, in new readings, meaning units that addressed the research questions were identified. These meaning units were then compared, sorted based on similar content, and further organised and clustered in alignment with the research questions. The analytical process was conducted independently by ES and HF in collaboration with MB and JB, and discussions were held until complete agreement was achieved. All authors provided approval for the final version.

### Ethical consideration

The study was approved by the University of Gothenburg ethical committee at the Institute of Health Care Science and followed the Helsinki Declaration [23]. Participants were notified that their involvement was entirely optional. They were assured of anonymity and were free to end the interview or discussion at any time without the need for an explanation.

## Results

The following quotes will be presented as FGD 1 and FGD 2, which refer to the two focus group discussions. The individual interviews will be referred to as 1–4.

### Implementation

The area of implementation illuminates feasibility by describing *how* the twinning initiative was delivered and *what* was delivered.

## Fidelity, dose, adaptations and reach: how the twinning initiative was delivered and achieved

The initial plan was for the midwives from GRMA and SBF to meet in person for a twinning launch following the first workshop, but due to the COVID-19 pandemic, all subsequent meetings were conducted online. Also, initial workshops focusing on developing strategic goals based on the MACAT were held virtually in Ghana with GRMA council members instead of in person. The participants described how the adjustment made it possible to maintain the planned start of the implementation process. Despite the obstacles to implementation posed by the COVID-19 pandemic, the participants reported that most activities were implemented according to plan, while others needed to be adapted.

One adaptation described by the participants involved shifting initiative funds from travel expenses towards purchasing internet modems. The purchase of modems facilitated virtual meetings between the associations and especially improved GRMA’s internal communication with regional chapters and GRMA council members. Initially, most GRMA council members were not used to virtual meetings but adapted to the new circumstances due to the twinning project.

Midwives were not really familiar with Zoom; even Internet connectivity wasn’t good, so the project enabled us to purchase modems for all the regional chapters. (1)

### Mechanisms of impact of the implementation

Shared experiences on strengthening both associations and increasing policy influence were driving the initiative forward. GRMA’s advocacy during COVID-19 highlighted this impact: initially, Ghana’s COVID committee excluded midwives, resulting in maternal and child healthcare gaps. Using the MACAT plan, GRMA advocated for midwives inclusion raising critical questions about maternal care, labor, postpartum care, and midwives' safety. The committee had overlooked these issues, leaving midwives without training, information, or protective equipment. A GRMA midwife noted, ‘*If midwives had been included in decision-making from the start, this wouldn’t have happened*.’ Once included, these gaps were addressed. SBF members found this experience insightful, as the Swedish midwifery association faced similar challenges. GRMA also strengthened leadership by setting structured plans with clear targets and facilitating regular board and regional meetings.

The participants from GRMA further explained that the association had been able to advocate at a higher health system level and had been invited to engage in discussions and petitions to establish midwifery leadership positions within the Ministry of Health.

The advocacy has been to push for midwifery to get a higher-level position at the national level and then also to get midwives represented when it comes to committees and other ad hoc meetings. (1)

During the initial MACAT assessment, the participants recognised that organising visits and online meetings with midwives in remote regions beyond the Greater Accra region would be a crucial method to enhance networking and coordination. These meetings occurred frequently and played a facilitating role in making plans to increase new membership rates, for example, through increased digitalisation of the membership process.

A critical challenge for GRMA was to increase its membership numbers. A higher membership rate would enable the association to advocate for better salaries and working conditions on a higher political level and increase its bargaining power, thereby making it a stronger association. Before the twinning initiative, midwives who wanted to become members had to print or collect a hard copy of the membership form, complete it, and either send it or deliver it to the association. As a result of the work within the project’s strategic plan, midwives could easily renew their professional pin certification, enabling them to practice legally without the cumbersome annual renewal process. A digitalisation process streamlined the membership application and made it more accessible and user-friendly, increasing membership numbers.

In line with the strategic plan, GRMA also improved the collection of dues from its members during the twinning. Instead of the previous method where regions collected dues and sent them to the national office, which was often difficult and time-consuming, they now deduct dues directly from members’ bank accounts when their salaries are deposited. The new way of working has streamlined the process, strengthening GRMA financially.

So, they are being deducted at source, and the deduction is paid into our account for us to disperse, so it has also made us, I would say, financially stronger. (4)

Another mechanism for change in line with the strategic plan was to recruit an administrative communication assistant at GRMA. The recruitment was seen to positively impact GRMA’s membership rates and the project’s visibility due to an increased presence on social media platforms.

I think visibility-wise, yes, the association has become visible in Ghana. Even in the remotest areas in Ghana, midwives get to know that the Ghana Registered Midwives Association is the association that speaks for midwives in Ghana. (FGD 1)

Lastly, a mechanism for change, highlighted by the participants, was organising online knowledge exchange seminars, which provided a meeting platform for members from the two associations. Through presentations and dialogue, researchers and clinical midwives from both countries could share experiences, information, and ideas and acquire new knowledge. Examples of discussion themes were ‘Experiences of midwives in managing obstetric emergencies’, ‘The transition from a professional organisation to a labour union’, and ‘Midwives’ work environment and ways to improve it’. The sessions were seen as fruitful and inspiring to both sides, and the knowledge was spread beyond the participants to other members, enhancing the twinning’s reach.

## Challenges related to the implementation of the twinning initiative

While the twinning was perceived mainly as positive, challenges were encountered along the way. The involvement of midwives from Sweden and Ghana was entirely voluntary, and several midwives grappled with unexpectedly time-consuming administrative tasks, leading to declining involvement. One midwife expressed a loss of motivation due to the extensive administrative workload diverting attention from the core midwifery aspects of the initiative. GRMA council members also highlighted issues with receiving funds on time, often experiencing delays that added stress about being able to carry out scheduled activities and meet reporting deadlines.

The project’s weakness was that funding didn’t come in, so sometimes you plan activities. Then we don’t have funding for it, so we have to wait for ages before the funding comes. (1)

## Usefulness of the twinning initiative

The twinning collaboration between the associations was described as useful in that it enabled new knowledge, inspiration, motivation, and increasing involvement among association members.

I think that even for the fact that we partner with Swedish midwives, it motivates midwives. You know, because for them, they want to see that, yes, we are not doing something in a vacuum, but we are benchmarking with better midwife associations or more experienced midwife associations to improve ourselves. (1)

Furthermore, the new partnership between GRMA and SBF enabled them to build confidence in bringing the two associations forward regarding visibility. GRMA increased its visibility during the initiative, which led to young midwives’ increased interest and participation within the association, while the SBF increased its visibility through blogs and published articles

You see, the main collaboration was about helping us stand strong and become visible. And I believe that we were able to achieve this, though not 100%, to a large extent. (4)

The twinning initiative was viewed by SBF participants as successful in raising awareness among Swedish midwives about beneficial midwifery practices and work methods outside Sweden, particularly in areas such as teamwork, independent work in smaller hospitals, and approaches to facilitating non-medical safe births. Joint Knowledge Experience seminars highlighted the importance of union membership in both countries as a platform for advancing the profession and fostering knowledge exchange. Additionally, midwives and students from both Ghana and Sweden expressed a strong desire for international work to deepen their collaboration and learning.

Our project has raised awareness about midwives in Ghana and in Sweden and have given us the opportunities to participate in national forums such as the Swedish Confederation of Professional Associations, we had not previously encountered. This demonstrates how, with modest resources, a project can be sanctioned, made visible, and gain access to forums where it wouldn’t otherwise have been present. This visibility fosters the development of networks and connects stakeholders who might not have met otherwise. (FGD 2)

## Outcomes of the implementation

The twinning initiative increased GRMA’s non-paying and paying members. Before the twinning period in 2020, GRMA had 412 non-paying members and 631 paying members. As the twinning initiative concluded in 2022, the association experienced a notable change, reaching 600 non-paying members and 1245 paying members – an increase of 46% and 97%, respectively. SBF shared insights from the twinning initiative through 14 blog posts, two articles in SBF’s *Jordemodern* magazine, and publications and outreach activities for *Union to Union* (a platform for international union solidarity) and *SACO* (the Swedish Confederation of Professional Associations). They also highlighted the international work of midwives based on the twinning initiative in community forums such as *Järvafestivalen* in Stockholm (a community-driven event for social and political engagement) and at the SBF Reproductive Health Conference. These efforts were believed to have enhanced Swedish midwives’ and the general public’s understanding of international midwifery practices while also underscoring the importance of continuing work like the twinning project to strengthen women’s and newborns’ health. Additionally, the three knowledge exchange sessions between Ghanaian and Swedish midwives and students were considered to have played a role in broadening perspectives on global midwifery practices and fostering the valuable exchange of ideas for strengthening midwifery labour unions in the two countries.

## Discussion

This study shows that the twinning initiative between the Swedish Association of Midwives and the Ghana Registered Midwives Association was successfully implemented regarding fidelity, dose, and reach, even though adaptations were made to the original project plan. GRMA and SBF increased their visibility via social media, blogs, and seminars, and GRMA also increased their visits to regional branches in Ghana. Two key outcomes of the implementation were the increase of new members of GRMA, which led to enhanced advocacy efforts and improved visibility and awareness of midwives’ roles in international contexts among Swedish midwives and students.

The study findings show that, despite the adaptations posed by the COVID-19 pandemic, the twinning successfully transitioned online, allowing for virtual workshops and strategic planning sessions. These results contrast with the midwifery twinning project between Tanzania and Canada [4], which noted online communication as a central challenge due to issues such as limited internet access and unreliable connectivity for midwives in Tanzania. One possible explanation for the success of online communication and organising online knowledge exchange seminars in our study is the reallocation of funds from travel expenses to purchasing internet modems and facilitating virtual meetings in place of in-person gatherings, which enabled GRMA to communicate with their members in the regional chapters.

Despite the generally positive perception of the twinning initiative between midwives in Ghana and Sweden, challenges similar to those reported in other midwifery twinning initiatives emerged [4,24]. The voluntary engagement of midwives from Ghana and Sweden resulted in time-consuming administrative duties, leading to decreased participation. These findings parallel those of Ireland et al. [24], who documented comparable experiences in the twinning programme involving midwives from Nepal and the United Kingdom. Improving the organisational and management skills within the profession demands a significant investment of time and effort [24]. Moreover, midwives involved in twinning typically serve as volunteers, balancing their involvement with full-time positions elsewhere, a pattern observed both in our study and in the research by Ireland et al. [24].

The strength of the twinning initiative lies in its capacity to implement change [25]. In the twinning collaboration between Ghana and Sweden, the mechanism of achieving impact and fostering change can be attributed to developing a structured plan with clearly defined targets and timelines for the twinning activities established at the outset of the initiative. Additionally, a notable finding is that GRMA enhanced its visibility and gained the ability to advocate for the importance of midwifery at a higher level within the healthcare system. GRMA’s enhanced advocacy ability is important as evidence shows that associations with the power to advocate are central to building partnerships and identifying opportunities within the public and private sectors to impact policymaking [26]. These findings align with the critical success factors for midwifery twinning programmes identified in a Delphi study by Cadée et al. [25].

Examining the implementation and factors influencing the association’s twinning using a structured evaluation framework [21] provided crucial insights into mechanisms for driving change and how the intervention operated in practice. Using a structured evaluation framework is also vital for building an evidence base to inform policy and practice [21], which is particularly important as twinning approaches, as exemplified by GRMA and SBF, can be ways to target global inequities in health by bringing together and empowering health professionals across cultures [5].

## Strengths and limitations

One key strength of this study lies in utilising the process evaluation guideline established by the Medical Research Council [12], which provided a structured approach to the evaluation. While the guideline offers advantages due to its comprehensiveness, it also presents limitations, as it was impractical to thoroughly address every aspect of the four main areas during data collection, potentially excluding valuable insights. To address this, the research team engaged in iterative discussions to identify the most relevant content in the four areas of the guidelines for the specific study. Another limitation of the study arises from the non-attendance of some invited participants. In combination with unreliable internet connectivity in Ghana, which affected the interview flow, these factors may have compromised the richness of the data. Nonetheless, upon analysing all interviews, recurring responses emerged, indicating that satisfactory conclusions were still reached despite challenges.

## Conclusion

The findings from the twinning initiative between the Ghana Registered Midwives Association and the Swedish Association of Midwives indicate an overall positive outcome despite deviations from the intended delivery due to the pandemic. The association from Ghana notably saw an increase in paying members, indicating a strengthening of the association. This suggests that twinning initiatives have the potential to enhance the capacity and presence of midwife associations, contributing to the advancement of women’s and newborn health as well as sexual and reproductive health and rights goals. The study underscores the importance of twinning initiatives in fostering knowledge exchange, forming new partnerships, and addressing healthcare quality challenges. The results offer valuable insights for other midwife associations considering similar twinning initiatives, highlighting potential challenges and benefits to guide future endeavours.

## Data Availability

The data sets used and/or analysed during the current study are available from the corresponding author upon reasonable request.

## Appendix 1– Interview guide

### Evaluation of a twinning project between midwife associations in Sweden and Ghana

#### Fidelity

(1) What is your overall experience of the entire project?

(2) Please describe what worked well in the project. How and why?

(3) Were any changes made during the project? If so, what were they and when were they made?

##### Dose

1. What did you expect to achieve from this project? In your opinion, to what extent was this achieved? Give an example.

##### Reach

(1) How do you assess your own engagement in the project? In which activities did you participate?

(2) Could your engagement have been improved? If so, how?

##### Acceptability

(1) In your opinion, what were the strengths of the project? Please give an example.

(2) What has been the easiest part in implementing the project? Please give an example.

(3) In your opinion, what were the weaknesses of the project? Please give an example.

(4) What has been the most challenging part in implementing the project? Please give an example.

(5) How could the project have been improved? Please give an example.

##### Mechanisms of Impact (how the project produces change)

(1) In your opinion, what have you achieved during the project? Please give an example.

-Increased visibility? If so, in what way?

-New partnerships? If yes, with whom?

-New memberships? If so, how, and in which forums?

(2) What factors contributed to the project’s achievements, and how did they do this?

-What were some obstacles to achievement?

(3) Has the project changed the way the association is working? If yes, in what way?
